# Patterns in outpatient antibiotic prescribing in Switzerland: a five-year analysis of claims data from 2019 to 2023

**DOI:** 10.1017/ash.2026.10862

**Published:** 2026-09-23

**Authors:** Philipp Stefan Aebi, Lena Zwicker, Melinda Toth, Caroline Bähler, Christian Frei, Maria Trottmann, Reto Auer, Adrian Rohrbasser

**Affiliations:** 1 Institute of Primary Health Care (BIHAM), University of Bern, Bern, Switzerland; 2 Department of Internal Medicine, Spital Thun, Thun, Switzerland; 3 Medbase Health Care Center, Wil, Switzerland; 4 Health Services Research, SWICA Health Insurance: SWICA Krankenversicherung AG, Switzerland; 5 Center for Primary Care and Public Health (Unisanté), University of Lausanne, Lausanne, Switzerland

## Abstract

**Objective:**

Antimicrobial resistance threatens the effectiveness of future therapies. Interventions to reduce the rate of unnecessary antibiotics prescription in the Swiss outpatient setting typically target primary care physicians (PCPs). However, patients might receive antibiotics from other healthcare providers. We set out to provide a descriptive analysis of total antibiotic prescribing and the providers who prescribed them among patients in such a plan.

**Methods:**

We performed a retrospective repeated yearly cross-sectional study from 2019 to 2023 in claims data from one insurer in Switzerland (SWICA insurance). We restricted the sample to patients registered in a managed-care plan with PCP serving as a gatekeeper in the SWICA database. We provide descriptive statistics of total antibiotic prescription fills, the proportion of antibiotics prescribed by the gatekeeping PCP vs other healthcare providers and the type of antibiotic prescribed.

**Results:**

The population comprised 106,307 patients in 2019 and 113,343 in 2023. The gatekeeping PCP accounted for 40% of antibiotic prescription in 2019, and 36% in 2023. In 2023, other group practices, other PCPs and hospital outpatient departments together accounted for 40% of prescription fills. Gatekeeping PCPs issued a higher proportion of Watch antibiotics than other providers in both 2019 (35% vs 25%) and 2023 (30% vs 21%).

**Conclusion:**

In a restricted sample of patients affiliated in a healthcare plan where PCP serve as gatekeepers in one insurance in Switzerland, most outpatient antibiotics were not prescribed by the primary gatekeeping PCP. Future antibiotic stewardship interventions in outpatients should consider further prescribers in the network.

## Introduction

In Switzerland, antibiotic resistance is a significant public health threat responsible for a rise in disability-adjusted life years, from 3,995 in 2010 to 6,805 in 2019.^1,2^ In 2015, the Federal Office of Public Health formally recognized the dangers of antibiotic overprescription, which increases the risk of antimicrobial resistance.^3–7^ In total ~80% of unnecessary antibiotic prescriptions are estimated to be written in outpatient settings,^8^ where most (allegedly 80%) antibiotics are prescribed by primary care providers (PCPs) for potentially self-limiting infections.^9,10^ Reducing over-prescription in primary care has become a key focus of antimicrobial stewardship interventions.

In Switzerland, PCPs are less likely to prescribe antibiotics than those in many other countries^11,12^, but they do continue to overprescribe antibiotics for common viral infections against which they are ineffective, or for self-limiting bacterial infections.^9^ In these cases, patients may ask for an antibiotic prescription or physicians may assume that their patients expect them.^13–16^ At least half of the prescriptions for potentially self-limiting diseases are considered avoidable.^9,17–19^ Apart from the total number of prescriptions, the type of antibiotic prescribed is central as broad-spectrum antibiotics contribute to the development of antimicrobial resistance.^20^

A recent study on health insurance claims data of CSS, a major health insurer in Switzerland, found that PCPs wrote proportionally fewer antimicrobial prescriptions than other healthcare providers in ambulatory care.^21^ This surprising finding requires validation, because these results may be influenced by the current PCP shortage in Switzerland, which causes people to seek help in various institutions, such as walk-in clinics or hospital emergency departments and therefore lowering the proportion of antibiotics prescribes by PCPs. By restricting analyses to patients in a managed-care health insurance, who have a registered responsible PCP, the so-called gatekeeper, we can test whether the lower than expected prescription by PCP compared to other ambulatory care settings such as specialized medicine or emergency care is also true when we can expect patients to have access to a PCP to get a prescription. If such analyses confirm that PCP are not the main prescribers of antibiotics in ambulatory care, this will justify targeting further healthcare professionals for antibiotics stewardship interventions.

We set out to provide descriptive analyses through a retrospective repeated cross-sectional study using anonymized claims data. We focus on trends in total antibiotic prescribing and the proportion of prescriptions by different healthcare providers. Additionally, we aim to analyze the type of antibiotic prescribed by different healthcare providers.

## Methods

### Study design and setting

We conducted a retrospective repeated cross-sectional study using health-insurance claims data from 2019 to 2023. We used data from SWICA health insurance, the fourth largest health insurer in Switzerland, which mainly serves the German-speaking region.^22^ The study population was restricted to individuals enrolled in a managed-care plan requiring consultation with their designated gatekeeping PCP before accessing specialist care or advanced diagnostic services.

The population was originally assembled for a subsequent intervention evaluation and included patients registered with participating practices and a weighted comparison population. Because the present study was descriptive baseline analysis, both groups were pooled for all analyses. The construction of the population, including the matching procedure, is described in Appendix 1.

### Outcomes and variable definitions

The primary outcome was the number of antibiotic prescriptions filled by patients in the outpatient setting. Secondary outcomes included the healthcare provider type responsible for each prescription, identified via the Zahlstellenregisternummer (ZSR), and the class of antibiotic prescribed, classified according to the Anatomical Therapeutic Chemical (ATC) system (J01, excluding J01Z).

We included the following patient-level variables: age; gender; insurance type; type of municipality they live in (rural vs urban); registered primary care provider (gatekeeper) and pharmaceutical cost groups (indicating long-term medication use). PCG is a classification system that categorizes individuals based on their pharmaceutical claims. The Swiss regulator uses it in risk adjustment, a redistribution system designed to promote fair competition and prevent risk selection.^23^

We categorized the prescribers by the following groups using their corresponding ZSR number: group practice of the gatekeeping primary care physician (PCP); other group practice (offices with mixed specialties); other general practitioners (including offices with multiple general practitioners); pediatricians (when in pediatricians only offices); urologists (when in urology only offices); gynecologists (when in gynecology only offices); other specialists; hospital outpatient.

Antibiotics were further grouped according to World Health Organizations (WHO) classification of antimicrobial use. ^24^

The data was collected as a yearly cross-sectional sample from 2019 to 2023. SWICA health researchers anonymized and provided our data sets.

### Statistics

We used descriptive statistics to report the antibiotic prescription fills, calculated corresponding proportions to assess the annual contribution of each prescriber group, and tested the difference between two proportions for significance using a *Z*-test. The primary analysis compared 2019, the last complete prepandemic year, with 2023, the most recent year available. This comparison was selected to reduce the direct influence of pandemic-related disruptions to healthcare use and antibiotic prescribing during 2020–2022. Annual trends across the complete study period from 2019 to 2023 are presented in Appendix 2.

Statistical analysis was performed using R software (version 4.3.1; R Foundation for Statistical Computing, Vienna, Austria). Graphs and visual representations were created using Adobe Illustrator (Adobe Inc., San Jose, CA, United States) and Microsoft Excel (Microsoft Corporation; Redmond, WA, United States) for clarity and presentation.

## Results

The pooled population comprised 106,307 individuals in 2019 and 113,343 in 2023. Of these, a fourth were children; most were between 19 and 65 years old, while 7%–8% were over 65 years old. Gender was equally distributed. Most patients lived in urban and peri-urban areas (90%). Most (92%) had no PCGs (Table 1). These patients were prescribed 24,601 prescriptions for antibiotics in 2019 and 26,479 in 2023.

**Table 1.** Demographic characteristics of the pooled analytical population, 2019 and 2023—full data for 2019–2023 can be found in appendix 3. Values are weighted n (%). PCG, pharmaceutical cost group. Weighted counts were rounded to the nearest whole number Table 1 long description.

Characteristic	2019 (*N* = 106,307)	2023 (*N* = 113,343)
**Age group 0–18**	24,641 (23.2%)	29,990 (26.5%)
**Age group 19–65**	74,722 (70.3%)	74,156 (65.4%)
**Age group > 65**	7,708 (7.3%)	10,016 (8.8%)
**Gender (male)**	55,272 (52.0%)	59,115 (52.2%)
**Urban municipality**	72,987 (68.7%)	77,136 (68.1%)
**Peri-urban municipality**	23,036 (21.7%)	24,599 (21.7%)
**Rural municipality**	11,047 (10.4%)	12,427 (11.0%)
**Number of PCGs = 0**	98,189 (92.4%)	102,390 (90.3%)
**Number of PCGs = 1**	6,940 (6.5%)	9,237 (8.1%)
**Number of PCGs = 2**	993 (.9%)	1,460 (1.3%)
**Number of PCGs ≥ 3**	184 (.2%)	256 (.2%)

**Figure 1.**
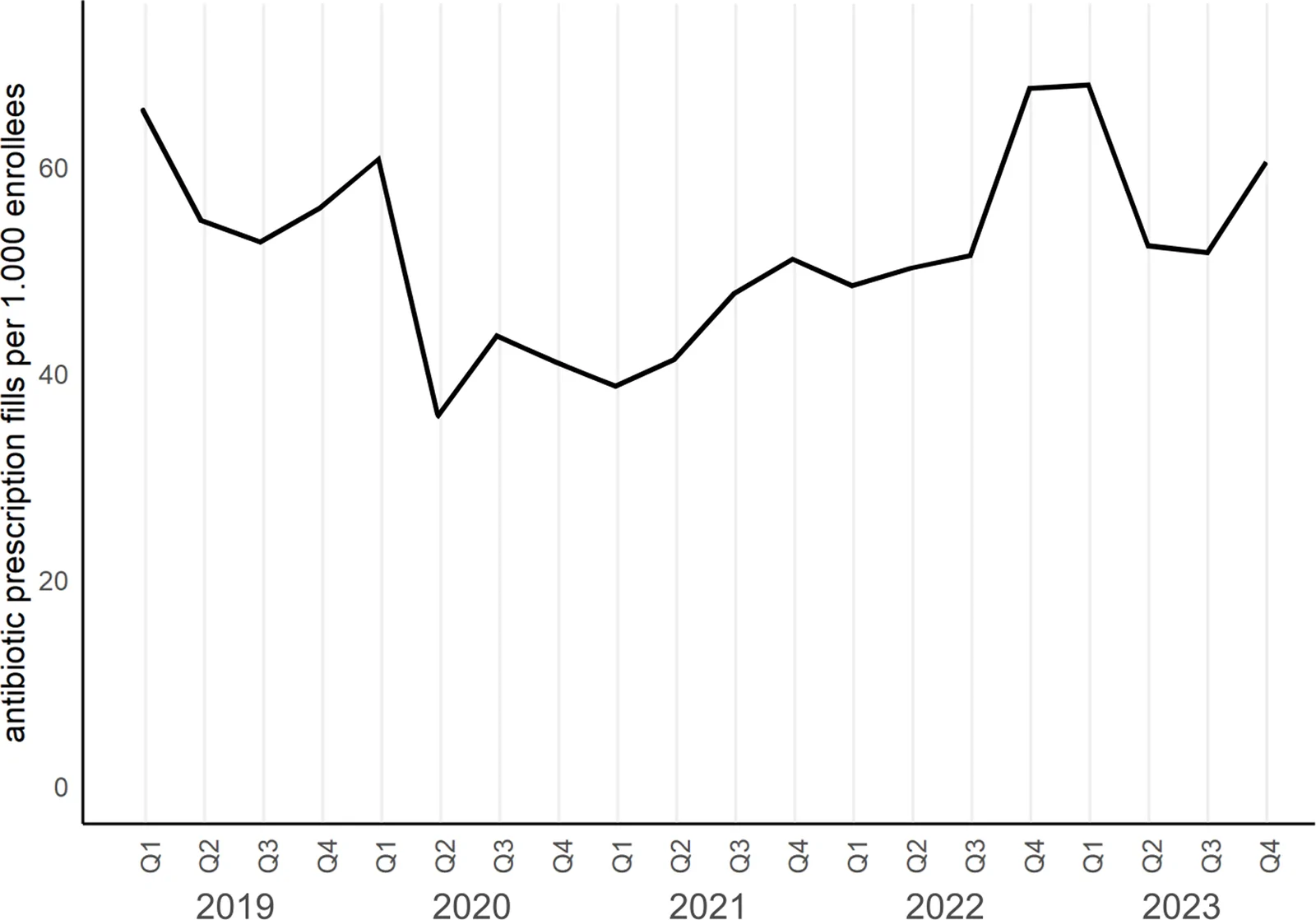
Change of antibiotic prescription fills per 1,000 enrollees from 2019 to 2023; Q1: first quarter of the year, Q2: second quarter of the year, Q3: third quarter of the year, Q4: fourth quarter of the year.

We observed a change in antibiotic prescription fill rate and prescriber groups over time. In our data set, we saw a decline in antibiotic prescription fills in 2020. After that, prescription fills increased, surpassing 2019 levels by 2023 (Figure 1).

**Figure 2.**
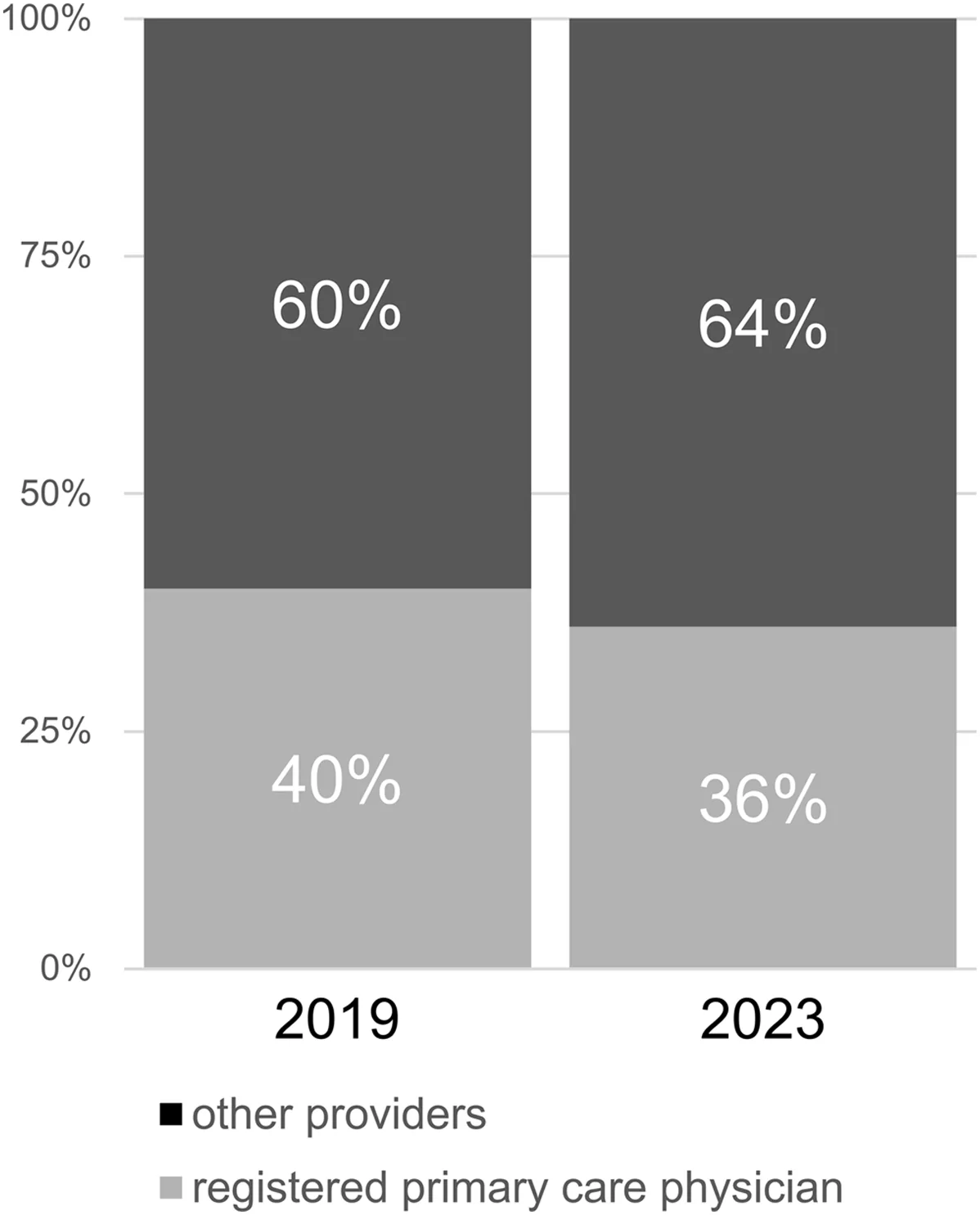
Rate of antibiotic prescription fills by prescriber in 2019 and 2023.

The registered PCPs had been responsible for 40% of antibiotic fills in 2019 but, by 2023, their share decreased to 36% while the percentage of other providers issuing antibiotic dispensations increased from 60% to 64% (*P* value < .001, Figure 2).

**Figure 3.**
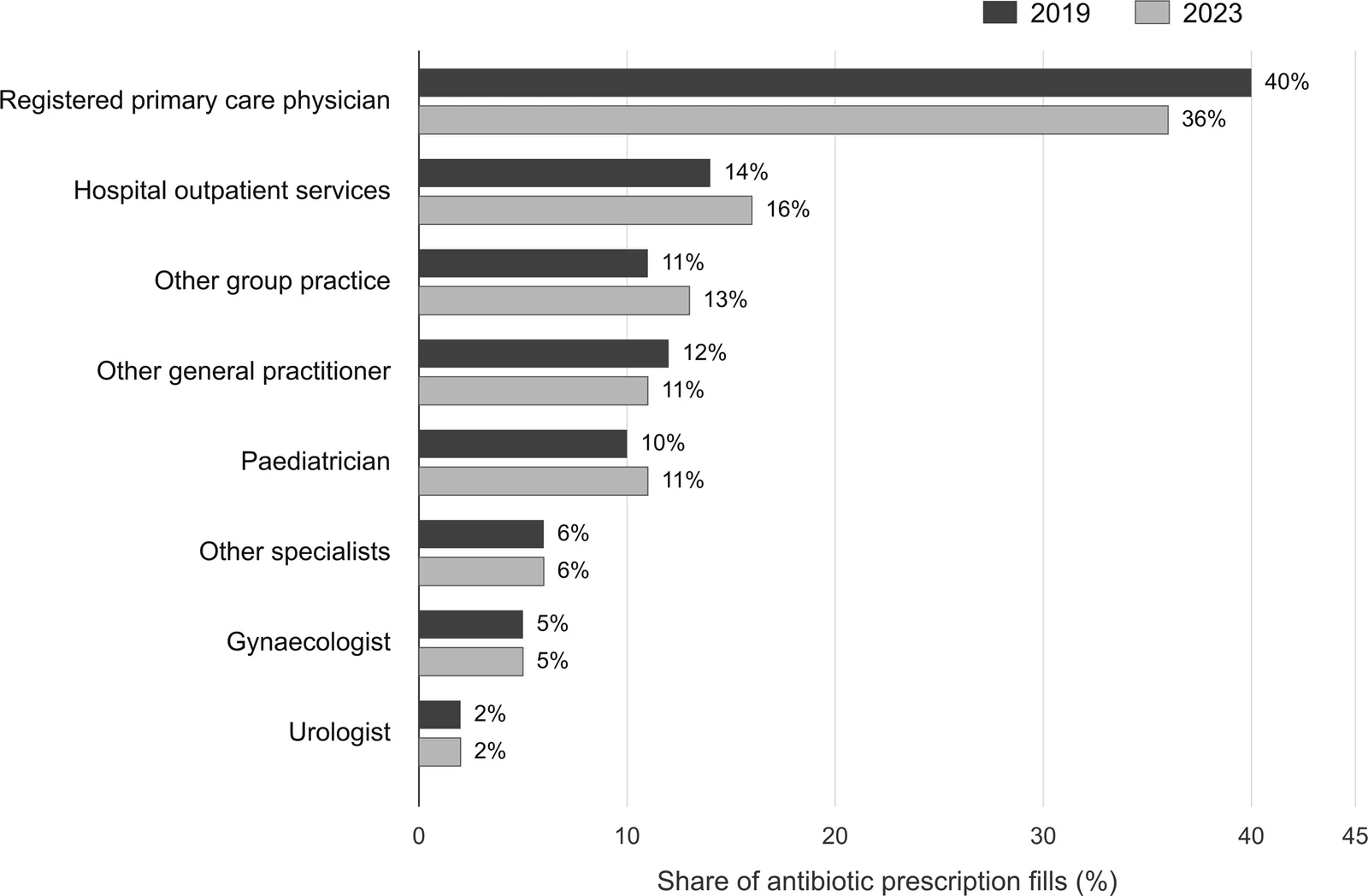
Antibiotic prescription fills by prescriber group in 2019 and 2023.

In 2019, other mixed-specialty group practices and general practitioners were responsible for approximately 23% of antibiotic dispensations, compared with 24% in 2023. Hospital outpatient services accounted for approximately 15% in 2019 and 16% in 2023. The proportions attributable to urologists (2%), gynecologists (5%), and other specialists (6%) remained largely unchanged (Figure 3).

**Figure 4.**
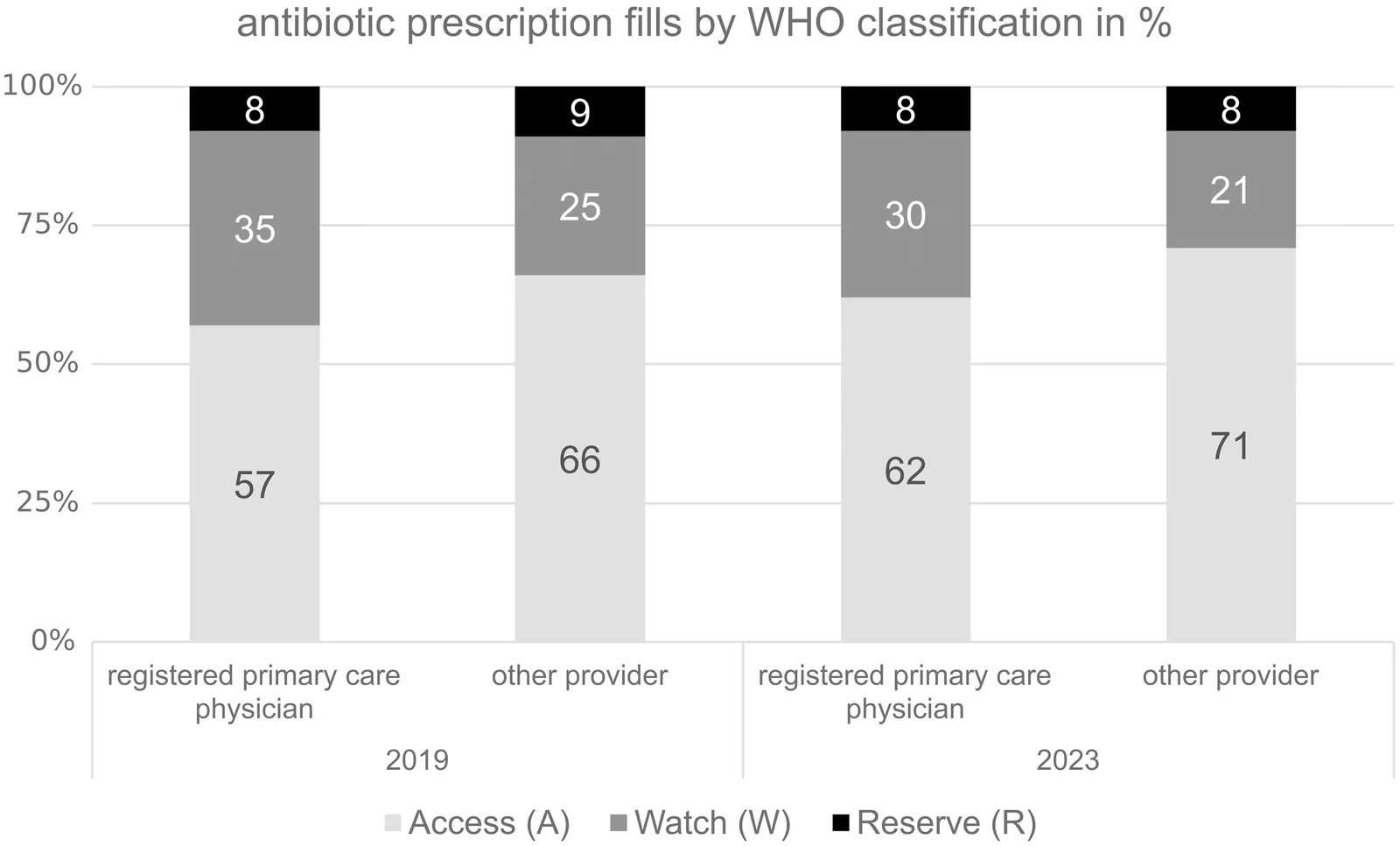
Antibiotic prescription fills by WHO classification in 2019 and 2023.

When we used the WHO’s guidelines and categorized the antibiotics by stewardship category, we observed shifts over time. In 2019, averages were 57% “Access,” 35% “Watch,” and 8% “Reserve.” In 2023, this shifted to 62% “Access,” 30% “Watch,” and 8% “Reserve.” For prescriptions issued by other physicians and hospitals, 2019 averages were 66% “Access,” 25% “Watch,” and 9% “Reserve.” In 2023, these proportions shifted to 71% “Access,” 21% “Watch,” and 8% “Reserve” (Figure 4).

## Discussion

Our main finding was a shift in outpatient antibiotic prescribing between 2019 and 2023. Although the total number of prescription fills increased, the proportion issued by registered PCPs decreased from 40% to 36%, while the contribution of other healthcare providers increased. Registered PCPs issued a higher proportion of Watch-classified antibiotics than other providers. Previous studies have highlighted that primary care is a significant contributor to outpatient antibiotic prescriptions, with 80% of such prescriptions being attributed to this sector.^8,10,25^ Although efforts have been made to inform PCPs of the risks of inappropriate prescribing ^6,26^, our findings show that a substantial portion of antibiotic use comes from other providers. The most recent analysis from data of another health insurer in Switzerland found a similar percentage of antibiotic prescriptions for general internal medicine doctors, which includes PCPs.^21^ Our study extends these findings by showing that substantial prescribing outside the registered PCP also occurs among patients enrolled in a managed-care plan who already have a designated gatekeeping PCP.

The decline in the proportion of prescriptions issued by registered PCPs coincided with an increase in total antibiotic prescriptions and a higher contribution from other healthcare providers. These concurrent trends do not establish that prescribing was displaced from registered PCPs to other providers or that they resulted from previous stewardship activities. Rather, they demonstrate that outpatient antibiotic prescribing is distributed across a broader care network, including other primary care practices, walk-in services, specialists and hospital outpatient departments. Interventions should also be designed to target in-hospital prescribers and walk-in practices that may see many patients with acute infections. Given the increasing shortage of PCPs in Switzerland,^27^ an important feature of our population was that all patients were enrolled in a managed-care plan and had a designated gatekeeping PCP. Therefore, the substantial proportion of antibiotic prescriptions issued by other healthcare providers is less likely to be explained solely by patients not having an established PCP. Nevertheless, registration with a gatekeeping PCP does not necessarily guarantee timely access to primary care and limited appointment availability may still have contributed to patients seeking care in other outpatient services.

Gatekeeping PCPs prescribed a higher proportion of Watch and a lower proportion of Access antibiotics than other healthcare providers, while the proportion of Reserve antibiotics was similar. The underlying reason remains unclear, and the available claims data cannot determine whether this difference reflects inappropriate antibiotic selection. Differences in infection type, patient characteristics or clinical case mix may contribute. To reduce the prescription of “Watch” antibiotics, antibiotic stewardship should focus not only on the appropriate timing of prescriptions but also on selecting the most suitable drug.^28^

Our study benefits from comprehensive data provided by SWICA, a major health insurance provider in Switzerland, which enabled a robust analysis of outpatient antibiotic prescribing patterns. The overall prescription rates from 2019 to 2023 were comparable to data gathered by the Swiss Federal Office of Public Health, which supports the validity of our data.^8^ The sharp decline in antibiotic prescriptions in 2020 is explained by the COVID-19 pandemic.^29^

Our study has several limitations. First, our study was conducted in the German-speaking region of Switzerland using claims data from a single health insurance company. Therefore, the findings may not be generalizable to other insurers or to Switzerland’s other language regions, where populations and prescribing practices may differ.^8,30,31^ Second, we only included patients who had a managed-care contract, so our study group includes a lower proportion of older adults than found in the general population. Third, we could not precisely identify the specialists in the “group practice” and “hospital outpatient” categories, so we could not determine which specialists were responsible for prescribing antibiotics within these settings. Finally, we decided not to calculate Defined Daily Doses (DDDs) as we were interested in the frequency of antibiotic prescription, rather than the dosage per use. However, a comparable analysis using DDDs showed a nearly identical distribution of prescriber categories when examining overall antimicrobial use.^21^

We found that the proportion of antibiotic prescriptions among healthcare providers shifted over time. Because the proportion of prescriptions issued by gatekeeping PCPs declined between 2019 and 2023, we suggest extending antimicrobial stewardship interventions to other groups of providers in both hospital and outpatient settings. Antibiotic stewardship in PCPs should additionally focus on reducing the number of broad-spectrum antibiotics prescribed.

## Supporting information

10.1017/ash.2026.10862.sm001 Aebi et al. supplementary material 1 Aebi et al. supplementary material

10.1017/ash.2026.10862.sm002 Aebi et al. supplementary material 2 Aebi et al. supplementary material

10.1017/ash.2026.10862.sm003 Aebi et al. supplementary material 3 Aebi et al. supplementary material

## Data Availability

The data sets used and/or analyzed during the current study are available from the corresponding author upon reasonable request.

## Supplementary material

The supplementary material for this article can be found at https://doi.org/10.1017/ash.2026.10862.

## Table 1. Long description

A table with 11 rows and 4 columns comparing demographic characteristics of a population in 2019 and 2023. The columns are labeled ‘Characteristic’, ‘2019 (N=106,307)’, and ‘2023 (N=113,343)’. The table includes data on age groups, gender, municipality types, and the number of pharmaceutical cost groups (PCGs). Row 1: Age group 0-18, 24,641 (23.2 percent), 29,990 (26.5 percent). Row 2: Age group 19-65, 74,722 (70.3 percent), 74,156 (65.4 percent). Row 3: Age group over 65, 7,708 (7.3 percent), 10,016 (8.8 percent). Row 4: Gender (male), 55,272 (52.0 percent), 59,115 (52.2 percent). Row 5: Urban municipality, 72,987 (68.7 percent), 77,136 (68.1 percent). Row 6: Peri-urban municipality, 23,036 (21.7 percent), 24,599 (21.7 percent). Row 7: Rural municipality, 11,047 (10.4 percent), 12,427 (11.0 percent). Row 8: Number of PCGs = 0, 98,189 (92.4 percent), 102,390 (90.3 percent). Row 9: Number of PCGs = 1, 6,940 (6.5 percent), 9,237 (8.1 percent). Row 10: Number of PCGs = 2, 993 (0.9 percent), 1,460 (1.3 percent). Row 11: Number of PCGs >= 3, 184 (0.2 percent), 256 (0.2 percent).

Navigate back to Table 1..
