# Supplementary material for: Patterns in outpatient antibiotic prescribing in Switzerland: a five-year analysis of claims data from 2019 to 2023
Source: Antimicrob Steward Healthc Epidemiol. 2026 Sep 23;6(1):e287. doi: 10.1017/ash.2026.10862 (PMC13760426; doi:10.1017/ash.2026.10862)
Supplement: Supplementary file 1 [file S2732494X26108626sup001.docx]

The study population was originally assembled for a planned intervention study evaluating the effects of a multimodal intervention to optimise antibiotic prescribing among PCPs. All 56 primary care practices affiliated with the Medbase Health Provider Group were invited by email to participate in the intervention, which was scheduled to begin in 2024. Within the SWICA claims database, the intervention population comprised patients enrolled in a managed-care insurance plan whose registered PCP worked at a participating Medbase practice. The comparison population comprised patients enrolled in a similar managed-care plan whose registered PCP was not affiliated with Medbase.

The comparison population was constructed using inverse probability of treatment weighting (IPTW), rather than individual matching. The probability of belonging to the intervention population was estimated using logistic regression based on age group, sex, municipality type, deductible category, premium subsidy recipient status and pharmaceutical cost group characteristics. Patients in the intervention population received a weight of 1, whereas patients in the comparison population were weighted by $P/\left( 1-P \right)$, where $P$represented the estimated probability of belonging to the intervention population. Because the study population constituted an unbalanced panel, with the number and composition of participants varying between years, weights were recalculated separately for each calendar year. Descriptive comparisons showed that differences in measured characteristics between the intervention and comparison populations were substantially reduced after weighting.

For the present descriptive baseline analysis, data from the intervention and weighted comparison populations were pooled. No comparisons of intervention effects were conducted because the intervention had not yet begun during the study period. The resulting dataset was intended to provide baseline data for a subsequent evaluation of changes in antibiotic prescribing after implementation of the intervention.
