# Supplementary material for: Patterns in outpatient antibiotic prescribing in Switzerland: a five-year analysis of claims data from 2019 to 2023
Source: Antimicrob Steward Healthc Epidemiol. 2026 Sep 23;6(1):e287. doi: 10.1017/ash.2026.10862 (PMC13760426; doi:10.1017/ash.2026.10862)
Supplement: Supplementary file 2 [file S2732494X26108626sup002.docx]

| **Table S1. Annual distribution of outpatient antibiotic prescription fills by prescriber group, 2019–2023** | | | | | | | | | | |
| --- | --- | --- | --- | --- | --- | --- | --- | --- | --- | --- |
| *Values are prescription fills per 1,000 insured persons and the percentage of all antibiotic prescription fills in the respective year.* | | | | | | | | | | |
|  |  |  |  |  |  |  |  |  |  |  |
| **Prescriber group** | **2019, rate per 1,000** | **2019, %** | **2020, rate per 1,000** | **2020, %** | **2021, rate per 1,000** | **2021, %** | **2022, rate per 1,000** | **2022, %** | **2023, rate per 1,000** | **2023, %** |
| Registered primary care physician | 92.1 | 40% | 72.0 | 40% | 64.8 | 36% | 77.1 | 35% | 84.1 | 36% |
| Other group practice | 25.8 | 11% | 20.5 | 11% | 21.1 | 12% | 29.4 | 13% | 31.3 | 13% |
| Other general practitioner | 27.1 | 12% | 20.3 | 11% | 19.9 | 11% | 24.4 | 11% | 25.4 | 11% |
| Paediatrician | 22.1 | 10% | 14.2 | 8% | 15.2 | 8% | 23.3 | 11% | 25.5 | 11% |
| Urologist | 4.4 | 2% | 3.9 | 2% | 4.4 | 2% | 4.9 | 2% | 4.6 | 2% |
| Gynaecologist | 11.5 | 5% | 10.2 | 6% | 10.8 | 6% | 11.0 | 5% | 11.1 | 5% |
| Hospital outpatient services | 33.1 | 14% | 28.3 | 16% | 30.4 | 17% | 35.6 | 16% | 37.5 | 16% |
| Other specialists | 13.7 | 6% | 12.6 | 7% | 13.2 | 7% | 12.8 | 6% | 13.5 | 6% |
| **Total** | **230** | **100%** | **182** | **100%** | **180** | **100%** | **218** | **100%** | **233** | **100%** |

| **Table S2. Annual distribution of outpatient antibiotic prescription fills according to the WHO AWaRe classification, by prescriber category, 2019–2023** | | | | | | | |
| --- | --- | --- | --- | --- | --- | --- | --- |
| *Rates are prescription fills per 1,000 insured persons; percentages are calculated within each prescriber category and year.* | | | | | | | |
|  |  |  |  |  |  |  |  |
| **Year** | **Prescriber category** | **Access rate** | **Access, %** | **Watch rate** | **Watch, %** | **Reserve rate** | **Reserve, %** |
| 2019 | Registered primary care physician | 53.2 | 57% | 33.3 | 35% | 7.6 | 8% |
| 2019 | Other providers | 92.8 | 66% | 35.0 | 25% | 12.0 | 9% |
| 2020 | Registered primary care physician | 44.2 | 60% | 22.4 | 30% | 7.1 | 10% |
| 2020 | Other providers | 73.8 | 66% | 26.9 | 24% | 11.1 | 10% |
| 2021 | Registered primary care physician | 40.3 | 61% | 18.9 | 29% | 6.9 | 10% |
| 2021 | Other providers | 79.6 | 68% | 25.7 | 22% | 11.8 | 10% |
| 2022 | Registered primary care physician | 47.4 | 60% | 23.9 | 30% | 7.1 | 9% |
| 2022 | Other providers | 101.5 | 71% | 30.1 | 21% | 12.1 | 8% |
| 2023 | Registered primary care physician | 53.0 | 62% | 25.3 | 30% | 7.1 | 8% |
| 2023 | Other providers | 107.1 | 71% | 31.9 | 21% | 11.9 | 8% |
|  |  |  |  |  |  |  |  |
