# Supplementary material for: Patterns in outpatient antibiotic prescribing in Switzerland: a five-year analysis of claims data from 2019 to 2023
Source: Antimicrob Steward Healthc Epidemiol. 2026 Sep 23;6(1):e287. doi: 10.1017/ash.2026.10862 (PMC13760426; doi:10.1017/ash.2026.10862)
Supplement: Supplementary file 3 [file S2732494X26108626sup003.docx]

| **Characteristic** | **2019 (N = 106,307)** | **2020 (N = 111,241)** | **2021 (N = 115,083)** | **2022 (N = 116,783)** | **2023 (N = 113,343)** |
| --- | --- | --- | --- | --- | --- |
| **Age group 0–18** | 24,641 (23.2%) | 26,821 (24.1%) | 28,836 (25.1%) | 30,159 (25.8%) | 29,990 (26.5%) |
| **Age group 19–65** | 74,722 (70.3%) | 76,925 (69.2%) | 78,235 (68.0%) | 78,078 (66.9%) | 74,156 (65.4%) |
| **Age group >65** | 7,708 (7.3%) | 8,459 (7.6%) | 9,006 (7.8%) | 9,535 (8.2%) | 10,016 (8.8%) |
| **Gender (male)** | 55,272 (52.0%) | 58,024 (52.2%) | 60,263 (52.4%) | 61,118 (52.3%) | 59,115 (52.2%) |
| **Urban municipality** | 72,987 (68.7%) | 75,991 (68.3%) | 78,589 (68.3%) | 80,027 (68.5%) | 77,136 (68.1%) |
| **Peri-urban municipality** | 23,036 (21.7%) | 24,194 (21.7%) | 25,037 (21.8%) | 25,315 (21.7%) | 24,599 (21.7%) |
| **Rural municipality** | 11,047 (10.4%) | 12,019 (10.8%) | 12,451 (10.8%) | 12,430 (10.6%) | 12,427 (11.0%) |
| **Number of PCGs = 0** | 98,189 (92.4%) | 102,319 (92.0%) | 105,097 (91.3%) | 106,334 (91.1%) | 102,390 (90.3%) |
| **Number of PCGs = 1** | 6,940 (6.5%) | 7,585 (6.8%) | 8,479 (7.4%) | 8,813 (7.5%) | 9,237 (8.1%) |
| **Number of PCGs = 2** | 993 (0.9%) | 1,146 (1.0%) | 1,280 (1.1%) | 1,418 (1.2%) | 1,460 (1.3%) |
| **Number of PCGs ≥3** | 184 (0.2%) | 192 (0.2%) | 227 (0.2%) | 219 (0.2%) | 256 (0.2%) |

Appendix 3. Demographic characteristics of the pooled analytical population, 2019–2023

Values are weighted n (%). PCG, pharmaceutical cost group. Weighted counts were rounded to the nearest whole number.
